# Contrasting Habitat Use at the Range Edge of an Endangered Grassland Specialist: Insights Into Plains‐Wanderer ( *Pedionomus torquatus* ) Habitat Use in a Periphery Population

**DOI:** 10.1002/ece3.71894

**Published:** 2025-08-10

**Authors:** Saskia P. Gerhardy, Steven Delean, Liberty G. M. Olds, Graeme Finlayson

**Affiliations:** ^1^ Department of Ecology and Evolutionary Biology The University of Adelaide Adelaide South Australia Australia; ^2^ Green Adelaide Adelaide South Australia Australia; ^3^ Bush Heritage Australia Melbourne Victoria Australia

**Keywords:** Australia, habitat selection, periphery populations, Plains‐wanderer

## Abstract

Periphery populations can use habitat that differs significantly from core populations, as these regions often represent altered ecological pressures and resource availability. We assess the habitat use of the endangered Plains‐wanderer ( *Pedionomus torquatus* ), a ground‐dwelling bird, in the periphery of the species' known distribution. Grasslands are key habitats in the species' core range, but these habitats are largely absent at the periphery of their range. Between 2022 and 2024, 29 Plains‐wanderers were tracked using VHF and GPS loggers to measure fine‐scale habitat use in the western range periphery. Plains‐wanderers used habitat primarily dominated by *Sclerolaena* species, averaging 55% of total cover and 11 cm in height, and the remainder was composed of 40% bare ground and 5% litter. The *Sclerolaena*‐dominated vegetation community used by Plains‐wanderers differed in overall composition compared to preferred habitat in the eastern core range, particularly in the absence of grasses, while the general habitat height structure and minimum required open ground remained similar to the core. This study identifies a novel vegetation association for the species, thereby extending the area of potential occupation to include a variety of open‐plains vegetation associations at the range edge. Our findings show that periphery populations of Plains‐wanderers exhibit greater ecological flexibility in habitat selection than previously recognised. As such, the long‐held notion that Plains‐wanderers are exclusively ‘grassland specialists’ should be broadened to incorporate the suitability of low forb‐like Chenopod shrublands. Localised studies of individual animal movement reveal the vegetation characteristics critical to the persistence of this threatened species at the edge of their range.

## Graphical abstract

Habitat use at the range periphery of an endangered, Australian endemic bird was found to differ from key habitat described in the core range. Our study identifies the long‐held notion that Plains‐wanderers are exclusively ‘grassland specialists’ should be broadened to incorporate low forb‐like chenopod shrublands. Notably, despite these differences in vegetation type, we observed that habitat height and structure remained similar between both regions. As such, we readdress whether this species should be considered a ‘grassland specialist’ and suggest that it is able to survive in a number of low, open‐plains habitats.

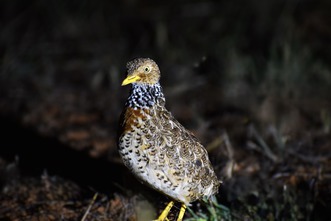

## 1 Introduction

Habitat selection theory states that individuals will select for environments and resources that improve their fitness (Rosenzweig 1981), with an individual's choice of habitat influenced by the availability, abundance and distribution of resources, in conjunction with the structure of the landscape within their distribution (Jenkins 1981; Willems and Hill 2009). However, resources are not homogenous across the landscape, and, when available, individuals will select for higher‐quality resources within a given range. These higher‐quality regions are generally selected for preferentially, relative to other habitats that might be available to the individual (Wiens 1984; Wainwright 1994).

Understanding this use of habitat is important in population studies, particularly at the periphery of a species range. Peripheral populations often face unique environmental pressures and may exhibit distinct ecological preferences compared to core populations (Channell and Lomolino 2000). Across a species distribution, areas with optimal habitats, often referred to as the core, are likely to contain the highest population density. Conversely, at the periphery of a species range, populations are expected to be smaller, fragmented, or restricted to smaller parcels of favourable habitat (Eckert et al. 2008). Populations on the periphery are typically restricted to less favourable habitat than the core and can therefore exhibit different habitat preferences (Eckert et al. 2008; Dudaniec et al. 2012). While often representing a smaller proportion of the whole population, periphery populations can be critical for species survival by providing opportunities for risk‐spreading or species refuge (Channell and Lomolino 2000). Additionally, peripheral populations may serve as important reservoirs of genetic diversity, offering the potential for resilience and adaptation (Hampe and Petit 2005). As such, a better understanding of habitat use across a species' marginal range can contribute to more effective habitat management and protection efforts, ultimately aiding in the recovery of endangered species across their entire distribution.

**FIGURE 1:**
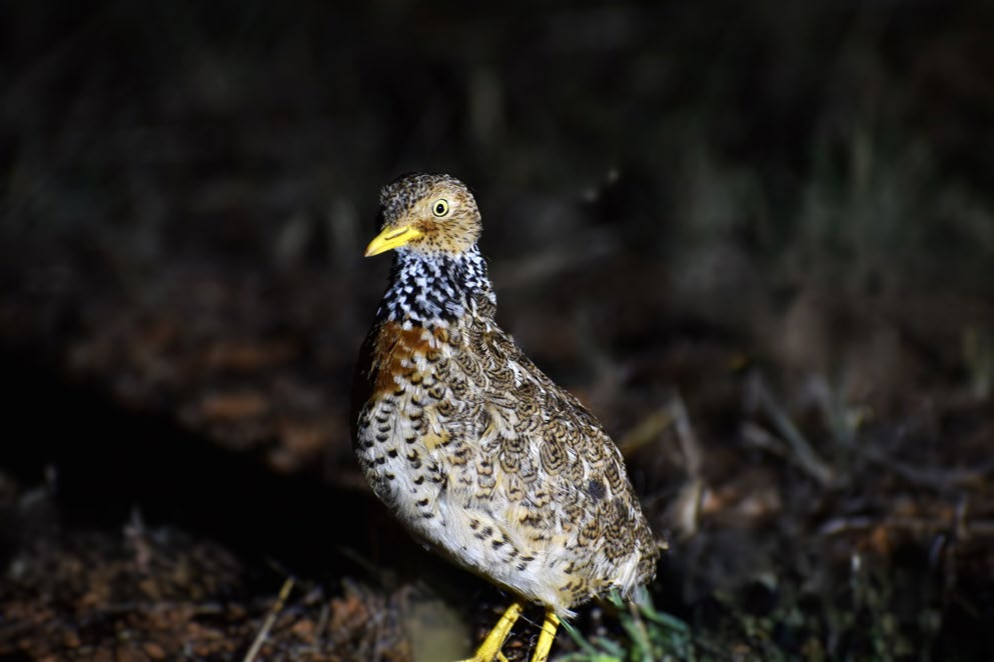
A female Plains‐wanderer ( *Pedionomus torquatus* ) showing a speckled neck pattern and buff patch on the upper breast.

**FIGURE 2:**
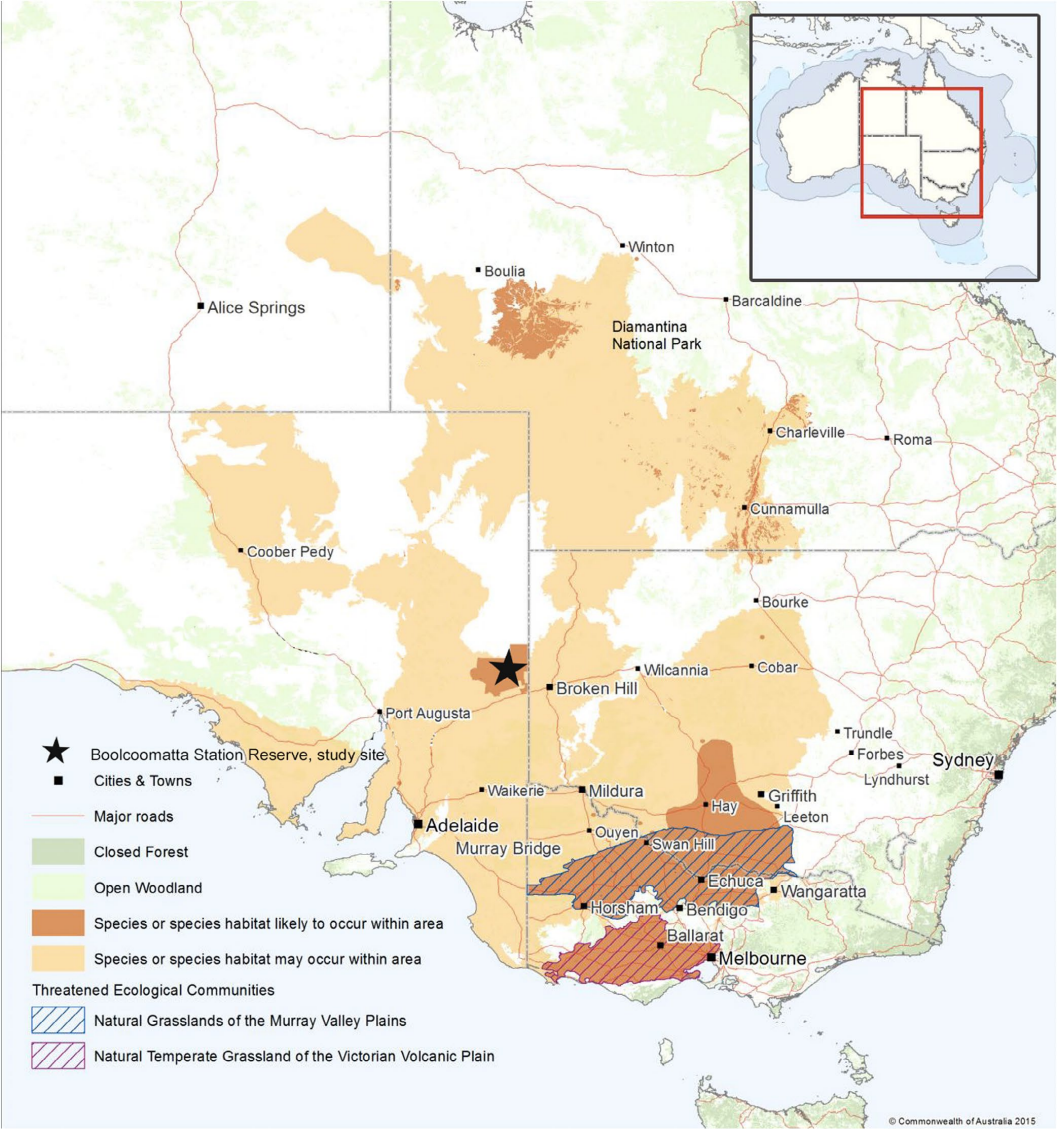
Current likely (brown) and historic (orange) Plains‐wanderer distribution in Australia. Map modified from the Commonwealth of Australia Plains‐wanderer Recovery Plan (2013) to reflect current understanding of population distribution. The star highlights the Boolcoomatta Station Reserve study site.

The ground‐dwelling Plains‐wanderer ( *Pedionomus torquatus* , Figure 1) is an Australian endemic and the sole extant species in its Family. It is considered a grassland specialist in the core of its range; however, habitat requirements in the range periphery are largely unknown. While once common throughout most of the grasslands in eastern Australia, the species is now restricted to a few fragmented populations in southern New South Wales, northern Victoria, southwestern Queensland and semi‐arid South Australia (Figure 2). The decline of the species has been attributed to significant habitat modification (Baker‐Gabb 1988; Baker‐Gabb et al. 1990, 2016). There has been a rapid reduction in the species distribution since European colonisation, with the species thought to be lost from much of its original range and current populations confined to fragmented remnants of preferred habitat (Bennett 1983). The Plains‐wanderer is now nationally listed as Critically Endangered under the Environment Protection and Biodiversity Conservation (EPBC) Act 1999 and Endangered under the IUCN Red List (BirdLife International 2022), with 250–5000 individuals estimated to be remaining throughout the former range (Commonwealth of Australia 2016, IUCN 2024). Current Plains‐wanderer distribution is now predominantly confined to two core populations in southeastern Australia (Figure 2). These are estimated to constitute 90% of the current known population and are priority regions for conservation efforts (Baker‐Gabb 1998; NSW National Parks and Wildlife Service 2002). The remaining 10% are considered to be in peripheral populations to the west and north of the core range. These populations are geographically separated primarily as a result of extensively modified pastoral land and have been assumed to be nomadic or declining remnant populations (Commonwealth of Australia 2016). There is little understanding as to whether the core and periphery populations are connected through unknown remnant populations or migratory routes. However, peripheral populations are thought to function independently of the core, with geographic and anthropogenic barriers making genetic exchange between the populations difficult (Commonwealth of Australia 2016).

In the core range, Plains‐wanderers prefer grasslands that are composed of approximately 40% native grasses, 50% bare ground and 10% litter, with a grass covering less than 10 cm in height (Baker‐Gabb 2016). Habitat structure is highly important, with birds avoiding areas of low (less than 10 cm) or high (greater than 30 cm) inter‐tussock space (Nugent et al. 2022). Furthermore, the species tends to avoid habitat with high levels of invasive plants or low levels of bare ground, as it reduces the openness between grass tussocks (Antos and Schultz 2020; Nugent et al. 2022). Critically, Plains‐wanderers are unable to persist in grasslands that are either excessively sparse or overly dense (Baker‐Gabb et al. 1990; Nugent et al. 2023). While these selective conditions have been observed in numerous studies (see Harrington et al. 1988; Baker‐Gabb 1990; Baker‐Gabb et al. 1990; Baker‐Gabb 1998; Parker 2009; Foreman 2010; Antos and Schultz 2020; DPIE 2020; Baker‐Gabb 2016; Nugent et al. 2022, 2023; Parker et al. 2024), vegetation preference has been quantified exclusively for populations in the core range, with much of the species' ecology remaining understudied in the western range limits within South Australia (Bennett 1983; Marchant and Higgins 1993). As the South Australian population represents the most western known distribution of Plains‐wanderer (Figure 2) and is significantly separated from populations in eastern Australia (approximately 500 km between the nearest known population), it is possible that differing ecological niches may be used by this population.

To determine whether there were ecological differences in a periphery population of Plains‐wanderers, we tracked individuals and quantified vegetation in used and available areas in the arid rangelands over a 24‐month period to: (1) determine the vegetation associations and habitat characteristics used by Plains‐wanderers in the South Australian semi‐arid rangeland; (2) assess whether the species is using specific habitat features or vegetation types within the broader available landscape; and (3) determine the key habitat structure to facilitate future monitoring efforts in areas with similar habitat types, enhancing the ability to detect the species across a broader range.

## 2 Methods

### 2.1 Study Site

Boolcoomatta Station Reserve (Figure 2), situated 100 km west of Broken Hill in the South Australian semi‐arid rangeland, is characterised by pastoral land use, with the vegetation assemblages heavily modified by previous and ongoing stocking regimes. Much of the remaining vegetation structure and composition within the region has been lost or severely altered as a result (Playfair and Robinson 1997). Remaining vegetation is dominated by low *Chenopod* shrubland, patchy grasslands and low woodlands on rocky ranges (Bellchambers and Baker‐Gabb 2006). Boolcoomatta Station Reserve was managed as a pastoral station for 150 years prior to Bush Heritage Australia, a not‐for‐profit environmental organisation, purchasing the pastoral lease in 2006 and managing the station as a conservation reserve. The reserve encompasses 65,000 ha of rangeland vegetation, primarily composed of low, open‐plains habitat (*Maireana* spp., *Atriplex* spp. and *Sclerolaena* spp.).

Rainfall averages 199 mm with high interannual variability (17–609 mm) and is a key factor in determining vegetation cover and species composition of the semi‐arid rangeland (Bellchambers and Baker‐Gabb 2006). Boolcoomatta Station Reserve experienced above‐average rainfall during 2022 (224 mm) and 2024 (239 mm), but below average in 2023 (140 mm; BOM (2024)).

### 2.2 Study Species

The Plains‐wanderer ( *P. torquatus* ) is a small ground‐dwelling bird that once occurred widely across southeastern Australia. The sexes are dimorphic, and, unlike most birds, females are larger and more boldly coloured than males. Plains‐wanderers measure 15–19 cm in height; males weigh 40–80 g, and females 55–105 g. Females are light brown in colour, with a black and white speckled collar and a rufous patch on the upper breast (Figure 1). Contrastingly, males only display the light brown plumage. Post‐hatching offspring care is paternal (Marchant and Higgins 1993). Plains‐wanderers are the sole representative from the Family *Pedionomidae* (Olson and Steadman 1981; Sibley et al. 1988). Owing to the species Critically Endangered listing (EPBC Act 1999) and genetic uniqueness, Plains‐wanderers have been recognised as one of the top five birds to save from extinction and are considered to be a global conservation priority (De Pietri et al. 2016; Jetz et al. 2014).

### 2.3 Plains‐Wanderer Surveys and Nest Detection

We conducted nocturnal surveys throughout open‐plains habitat in Boolcoomatta Station Reserve between September 2022 and May 2024 to detect the occurrence of Plains‐wanderers and Plains‐wanderer nest sites. Surveys began one hour after sunset from a slow‐moving vehicle (8–10 km/h) using a thermal camera to scan the landscape, alternating on either side of the vehicle. Surveys were conducted nocturnally, as this provides a higher contrast between highly cryptic birds and the surrounding habitat, allowing for better detection than diurnal surveys (Baker‐Gabb et al. 1990, 2016; Dawlings et al. 2024). Off‐road transects were driven in parallel approximately 60 m apart (the furthest distance a Plains‐wanderer can be reliably detected with a thermal camera is 30 m (Dawlings et al. 2024)). In total, we surveyed 619 km of transects within areas where Plains‐wanderer had previously been recorded, along with areas of habitat deemed potentially suitable for the species (i.e., excluding areas with high tree cover). During the first survey period in Spring 2022, this involved exploratory surveys across 7680 ha of Boolcoomatta Station Reserve in vegetation associations including *Sclerolaena divaricata, Maireana sedifolia, Maireana aphylla, Maireana astrotricha, Atriplex vesicaria* and *Chenopodium nitraraceum*. Five regions of confirmed Plains‐wanderer sightings were determined, with permanent monitoring sites subsequently established in each of these and monitored twice every six months. These sites were the only regions in the property where Plains‐wanderers were detected and were established as permanent sites to ensure efforts focused on regions with confirmed species presence. The sizes of the sites were variable, ranging between 109 and 381 ha (average = 249.8 ha, standard deviation = 12.2 ha), with the site size influenced by the extent of similar habitat in that region. To allow for the difference in size, the total number of birds observed per site was compared with the total distance (in km) traversed per site to calculate an encounter rate.

Detectability with the thermal camera was impeded in more densely vegetated areas. Consequently, five Song Meters (SM4 Wildlife Acoustics) were also deployed between Spring 2022 and Spring 2023 at sites where Plains‐wanderers were not detected during nocturnal surveys. Vegetation associations where devices were placed included *Maireana pyramidata, Maireana astrotricha* and *Atriplex vesicaria* , as well as areas that had experienced significant rainfall and had subsequent high levels of vegetation growth (i.e., dense swathes of *Eragrostis* sp. in washout areas). Previous studies suggested that the maximum distance in which Song Meters could detect Plains‐wanderer calls was 180 m (Rowe and Balasubramaniam 2020); hence, all Song Meters were placed at least 350 m from the nearest known Plains‐wanderer population to account for uncertainty in home‐range sizes within this region and the potential for overlapping radiuses. Each device was placed 1 m off the ground and was programmed to record for one hour at both sunrise and sunset, when Plains‐wanderer calls are easiest to detect (Rowe et al. 2023). Recordings were analysed using the ARBIMON (Aide et al. 2013) web‐based platform using a Plains‐wanderer recognition file provided by the Plains‐wanderer Recovery Team (Rowe et al. 2023). Song Meters placed in vegetation communities where Plains‐wanderers were not detected during nocturnal surveys similarly did not detect any Plains‐wanderer calls, suggesting these regions were not used by the species during the study period.

### 2.4 Radio and GPS Tracking of Plains‐Wanderers

Plains‐wanderers observed on nocturnal surveys were captured using a hand‐held net and fitted with an Incoly leg band issued by the Australian Bird and Bat Banding Scheme (ABBBS), which was placed on the bird's left tarsus. Standard morphometric measurements (age, sex, weight and moult) were taken from all captured birds (Lowe 1989). Birds over 90 g were fitted with a 1.2 g GPS receiver (Pinpoint 10, SWIFT fix strategy, Lotek, UK) coupled with a 0.6 g VHF transmitter (PicoPip Ag317, Lotek, UK) (< 3% of body mass). Birds weighing 45–90 g were fitted with a single 0.6 g VHF transmitter (PicoPip Ag317, Lotek, UK). Both tracking devices were attached via a backpack harness constructed with a size 12 fitted elastic band (Nugent et al. 2023). At the end of the study period, tracked birds were recaptured, the GPS and VHF devices removed, and the data from the GPS units downloaded.

Three field trips were conducted between Spring 2022 and Spring 2023 for six weeks each, where a total of 29 birds were fitted with a tracking device. All males (*n* = 10) fell under the weight limit for the coupled tracking devices and were therefore tracked manually using a single VHF. Females (*n* = 19) were tracked using the coupled VHF and GPS devices. In total, 28 trackers were collected, or at least the data was downloaded, with a single bird lost from the study region, likely predated (Appendix 1).

**FIGURE 3:**
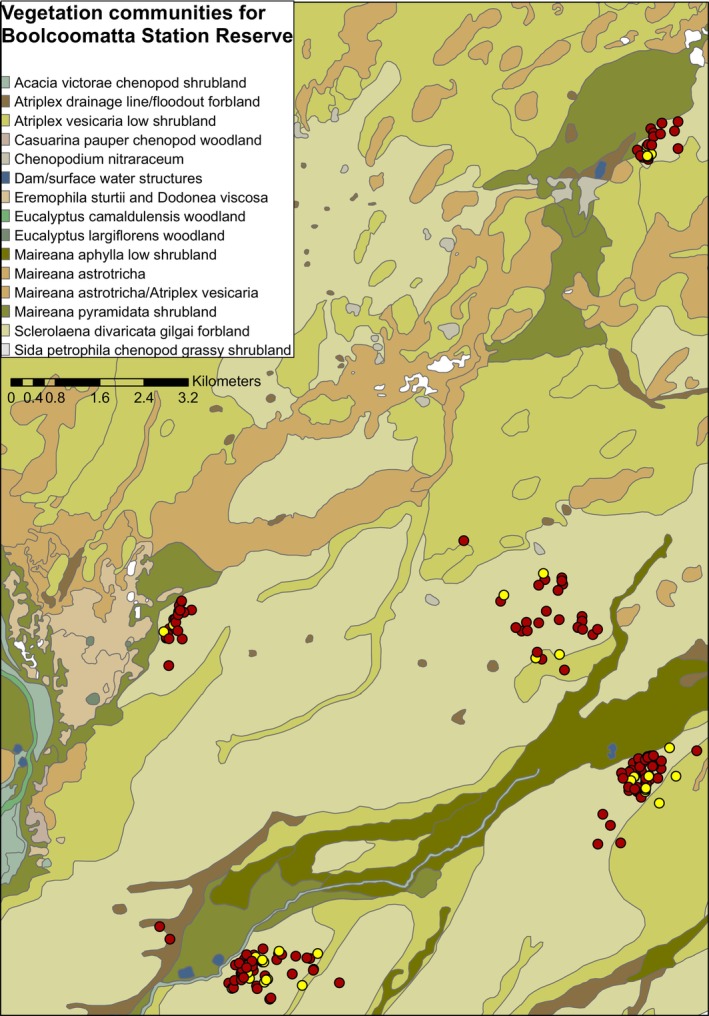
Vegetation communities of Boolcoomatta Station Reserve with total Plains‐wanderer sightings (red) and nest sites (yellow) between August 2022 and June 2024. Of the 308 bird and nest sightings, 295 observations were made in *Sclerolaena divaricata* associations (sage green), 2 in Atriplex drainage lines (dark brown), 10 in *Atriplex vesicaria* shrublands (lime green) and 1 in *Maireana pyramidata* associations (dark green). Vegetation map provided by Bush Heritage Australia.

GPS receivers were scheduled to record GPS fixes four times a day at 4‐hourly intervals from dawn to dusk (6.00, 10.00, 14.00 and 18.00) and remained on the birds for 20–30 days. Manual VHF observations involved tracking the birds to within 5 m of their locations at least every 2 days for a 30‐day period. We chose not to track the birds daily to reduce disturbance and to minimise potential influence on the birds dispersing from the area. All GPS locations of Plains‐wanderers detected during the survey periods were overlaid on existing vegetation maps of the property using ArcGIS 10 (ESRI 2011) to determine habitat associations of Plains‐wanderer presence (Figure 3).

### 2.5 Home‐Range Analysis

The extent of the home range of each tracked bird was determined using a Minimum Convex Polygon (MCP) encompassing all fixes of the individual. Home ranges were determined for each individual to assess the spatial extent of areas surveyed to measure vegetation compositions. During this study, two birds were recorded with a highly overlapping MCP. To avoid duplicating habitat data, we combined the two overlapping MCPs into a single polygon. As we aimed to examine the fine‐scale habitat use of Plains‐wanderers rather than focusing on individual habitat preferences, this approach was deemed appropriate for our study. In total, 27 MCPs were created and were considered an area of ‘used’ habitat.

To determine whether the full extent of individual home ranges was reached, the daily MCP home‐range size was measured and plotted against the number of tracked days. By plotting the curve and assessing whether it reached an asymptote, it could be determined whether MCPs reflected the full size of a projected home range (Harris et al. 1990).

To estimate effective home‐range size and activity centres, kernel density estimates were determined for each individual (Worton 1989). Unlike MCPs, kernel density estimates provide a classification of regions from highest to lowest use, providing a more comprehensive understanding of how an individual uses a home range. For this study, we considered two kernel levels: 50% kernel density representing the central activity centre, often where nest sites are located, and 95% kernel density representing the total area of the individual's home range (Samuel et al. 1985; Kie et al. 2010). Average home‐range sizes and standard deviation (SD) were determined across tracked birds. Trackers used for females returned coordinate fixes at higher temporal frequencies than for males, permitting exploration of continuous time movement models to estimate autocorrelated kernel density estimates (AKDE), which account for temporal autocorrelation in movement data. This approach can yield different estimates of the area used by individuals when there is autocorrelation in the movement process compared to KDEs that assume independent coordinate locations. However, the robustness of the AKDE estimates depends on having adequate effective sample sizes and on the assumptions of the autocorrelation error model being met. AKDE estimates were calculated using the ctmm (ctmm‐1.2.0; Fleming and Calabrese 2022) in the program *R* (version 4.2.2, R Core Team 2022).

### 2.6 Paired Survey Design

**FIGURE 4:**
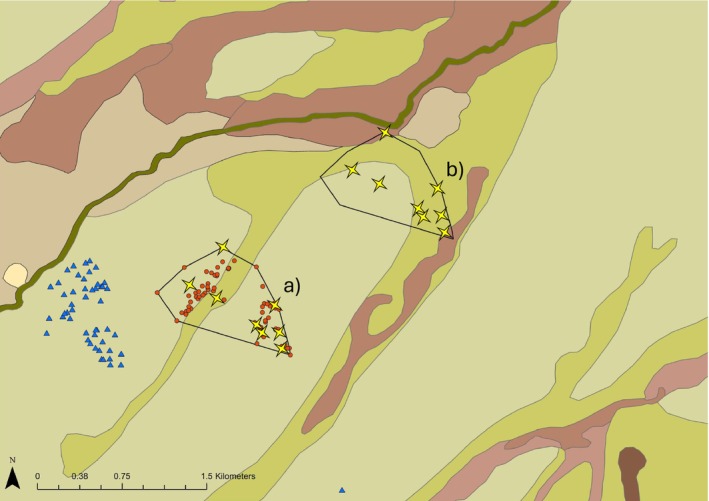
Paired polygon design showing a) the GPS points collected for a single bird (red dots) during the GPS tracking period and the estimated home range generated through a minimum convex polygon (MCP), and b) the paired polygon placed ~2 km away from the tracked bird's home range in an area that was not known to be used by other Plains‐wanderers. Blue triangles represent sites where other Plains‐wanderers had been recorded during the study period. Yellow stars in both (a) and (b) indicate sites where vegetation surveys were conducted. Points are overlaid on the vegetation layer where sage green = *Sclerolaena divaricata*, lime green = *Atriplex vesicaria* , brown = *Chenopod* drainage lines, dark green = flood zones and light brown = *Maireana aphylla* and *Maireana pyramidata* sand plains.

To determine whether Plains‐wanderers used a particular vegetation structure, a paired survey design was established. MCPs of ‘used’ habitat were visualised on GIS (ArcGIS 10, ESRI 2011), and eight GPS fixes from the tracking period were randomly selected as ‘used’ sites within each MCP. For each ‘used’ MCP, a paired polygon of equal size and dimension was randomly placed 1.5–2.5 km away (*n* = 27) from the used MCP and in an area in which Plains‐wanderers were not observed throughout the tracking period, nocturnal surveys, or acoustic surveys (Figure 4). This paired polygon represented habitat that was ‘available’ to Plains‐wanderers but was not known to be used during the time of study. Selection of ‘available’ polygons followed systematic selection guided by predefined exclusion criteria; locations were avoided if other Plains‐wanderers were detected in a polygon either through tracking or nocturnal surveys, or if they included ecologically unsuitable features for the species (i.e., tall trees, dams, roads, etc.). In instances where multiple candidate areas met these criteria, the polygon was incrementally distanced from the ‘used’ polygon until a site met all criteria. The ~2 km distance was chosen as it represented a larger distance between home ranges than recorded in previous studies (approximately 300–450 m diameter) (Baker‐Gabb et al. 1990; Commonwealth of Australia 2016), reflected a greater distance travelled than what was recorded in this study (a maximum of 800 m), and accommodated for potential overlap with the home ranges of other Plains‐wanderers present within the area. While ‘available’ MCPs were thoroughly surveyed for Plains‐wanderers, it is possible that undetected birds may have been present during the time of surveys. However, this is deemed unlikely, as these sites were intensely surveyed through both the nocturnal and the acoustic monitoring.

### 2.7 Vegetation Surveys

Eight vegetation surveys were conducted for each tracked Plains‐wanderer within the ‘used’ MCP, no more than a week after the tracking period. Due to the heterogeneous vegetation within Boolcoomatta Station Reserve, we found the ‘golf ball method’ (Schultz et al. 2017), a 1 × 1 m quadrat typically used for identifying ‘suitable’ Plains‐wanderer habitat, to be an unsuitable technique for classifying vegetation. Instead, we conducted vegetation surveys using a modified AusPlot method (White et al. 2012), whereby a 20 × 20 m grid was placed over a randomly selected GPS waypoint (either from the GPS logger or manual tracking data). This allowed for a wider range of habitat to be assessed per survey and allowed for GPS inaccuracy (up to 5 m). For this study, 20 m transect lines were run along the 5, 10 and 15 m intervals along both the *x* and *y* axes, so there were a total of six 20 m transect lines per vegetation survey. At each 1 m point along the transect line, a record was made of the plant species and height, with a total of 126 intercept points for each AusPlot survey.

For each intersect, the following vegetative features were recorded: bare ground/gravel, litter, cryptogamic crust, forbs (native and invasive), Chenopods, grass, vegetation height and dead vegetation. The term ‘Chenopod’ was used to refer to all *Chenopodiaceae*, except the genus *Sclerolaena*, which was recorded separately as it was the most dominant vegetation type recorded at the time of surveys and exhibited a largely short, sprawling growth type compared to the woodier growth type of other local Chenopod species (namely *Maireana* species). Vegetation was considered dead when it was dry, brittle and had lost significant leaf cover but was still attached to the rooting body. Vegetation that was detached from a rooting body was considered litter. Vegetation composition, height and percent cover were then determined for each 20 × 20 m AusPlot of used vegetation. Vegetation surveys were then repeated within an ‘available’ polygon, using an identical array of random survey sites in relation to the ‘used’ MCP (Figure 4).

### 2.8 Comparison of Used Versus Available Habitat

The differences in habitat structure between areas ‘used’ by Plains‐wanderers and those that were ‘available’ were visualised using non‐metric multidimensional scaling (nMDS) using the metaMDS function from the vegan package (vegan 2.6‐4; Oksanen et al. 2007) in *R*. The analysis included nine habitat structure variables: *Sclerolaena* cover, *Chenopod* cover, native forb cover, dead vegetation cover, invasive forb cover, native grass cover, bare ground cover, litter cover and vegetation height. A Euclidean distance was used, and the configuration was limited to four dimensions to capture the complexity of the habitat relationships.

### 2.9 Factors Affecting Habitat Use by Plains‐Wanderers

To estimate the optimal understory vegetation height range that is used by Plains‐wanderers, we modelled the relationship between the probability that habitat is used and the mean vegetation height using a generalised linear mixed effects model (GLMM) with the *glmmTMB* package (glmmTMB‐1.1.11; Brooks et al. 2017) in *R*. To account for variability in the mean probability of habitat use between individual birds, we included a random intercept term in all models. This term was adjusted for individual birds being sampled across multiple polygons. The model was fitted with *used* vs. *available* habitat as the response and mean height of vegetation in the quadrat as the explanatory variable.

Previous studies have shown that Plains‐wanderers use habitat where bare ground is approximately 50% of the survey area (Baker‐Gabb 2016). To determine the optima for the amount of bare ground that is used by Plains‐wanderers in the semi‐arid rangeland, we modelled the relationship between the probability that habitat was used and the average percent cover of bare ground. We fitted *used* vs. *available* habitat as the response and average percentage of bare ground as the explanatory variable.

We hypothesised that Plains‐wanderers would use habitat with specific vegetation compositions, including low native grasses interspersed with substantive areas of bare ground. Vegetation structure was quantified using percent cover estimates from line‐point intercept transects, including the following habitat categories: *Sclerolaena* cover, Chenopod cover, native forb cover, dead vegetation cover, invasive forb cover, native grass cover, bare ground cover, litter cover and vegetation height. As these variables are compositional (sum to 100%), they are interdependent and cannot be included collectively as explanatory variables in a model. To address this, we applied log‐ratio transformation to the compositional data (Greenacre 2021), converting percent cover into pairwise log‐ratios.

To retain focus on the ecological interpretation of the transformed variables, we used weighted stepwise selection of pairwise log‐ratios between the vegetation categories to identify the log‐ratios that explained the greatest variation in vegetation composition (Greenacre 2019). Weights were determined by the variance of the log ratio.

GLMM were fitted using each log ratio independently, and these models were ranked using Akaike Information Criterion with small‐sample bias correction (AIC_c_) along with a null model in the candidate set to identify the log ratio variables with the highest relative importance. Stepwise forward selection of log ratios using AIC_c_ identified the subset of log ratios that best predicted the probability the habitat was used. Although stepwise methods may be criticised for risks of overestimating goodness of fit, we use them here to reduce the dimensionality of the number of potential predictor combinations and simplify interpretation (Greenacre 2019), which was also guided by the independent analysis of log ratios. Principal component analysis (PCA) was used to visualise correlations among the selected log ratios and to explore groupings between used and available habitat.

To enhance interpretability of the results, we also constructed models for the probability habitat was used that included the vegetation categories (*Sclerolaena* cover, Chenopod cover, native forb cover, dead vegetation cover, invasive forb cover, native grass cover and litter cover) separately as explanatory variables. In all models, bird identification was a random effect, accounting for variability between individual Plains‐wanderers. We used residual diagnostic plots to test for a nonlinear relationship of habitat use and used natural splines with 2°–4° of freedom to determine the required smoothness (based on AIC_c_ model rank). For this analysis, the relative cover of *Sclerolaena* was the only variable where the model fit was improved by including a spline term to account for nonlinearity.

We assessed whether the average vegetation height in used and available habitats was influenced by species composition. To do this, we modelled the average height of each vegetation category as the response variable against the ‘used’ versus ‘available’ habitat classification. These models were fitted separately as linear mixed effects models (LMM), with bird identification included as a random effect. Diagnostic checks, including residual analysis, were performed to ensure model adequacy, and pseudo‐*R* ^2^ metrics using the MuMIn package (MuMIn‐1.47.1; Bartoń 2022) were included to assess model performance. To rank the models, we used AIC_c_.

## 3 Results

### 3.1 Trends in Vegetation Community Selection

From September 2022 to May 2024, we detected 211 adult Plains‐wanderers and 61 dependent chicks at Boolcoomatta Station Reserve. Of the 272 Plains‐wanderer sightings, 260 birds were identified in *Sclerolaena divaricata* gilgai forblands, with the remaining birds identified on the fringes of this association in either *Maireana pyramidata* shrubland (*n* = 1) or *Atriplex vesicaria* drainage lines and shrublands (*n* = 11) (Figure 3). Of the 36 nest sites detected, 35 were in *Sclerolaena divaricata* associations and one in an *Atriplex vesicaria* shrubland (Figure 3).

### 3.2 Home‐Range Analysis

The mean home‐range size for Plains‐wanderers (*n* = 27) at Boolcoomatta Station Reserve was 53.6 ha (SD = 52.0 ha) calculated using 95% Kernel Density Estimate (Appendix 2). Males appeared to have slightly larger home ranges (*n* = 9, avg. = 71.6 ha (SD = 63.8 ha)) than females (*n* = 17, avg. = 47.2 ha (SD = 44.3 ha)), noting there were methodological differences between the two sexes. Two individuals had highly overlapping home ranges and were considered to be a breeding pair during the period. During the nesting and egg‐laying period, paired birds were found within 2–10 m of each other and only occupied an area of 1.0 ha. However, once incubation commenced, the female was recorded moving up to 1 km from the nest site and did not return to the males' home range. During the study period, the males remained within the 1 ha MCP, but the females' total home range encapsulated 24.8 ha. Home‐range size generally peaked at day 16, with all birds except for four females reaching a stable asymptotic home‐range size (Appendix 3).

### 3.3 Plains‐Wanderer Habitat Selection

**FIGURE 5:**
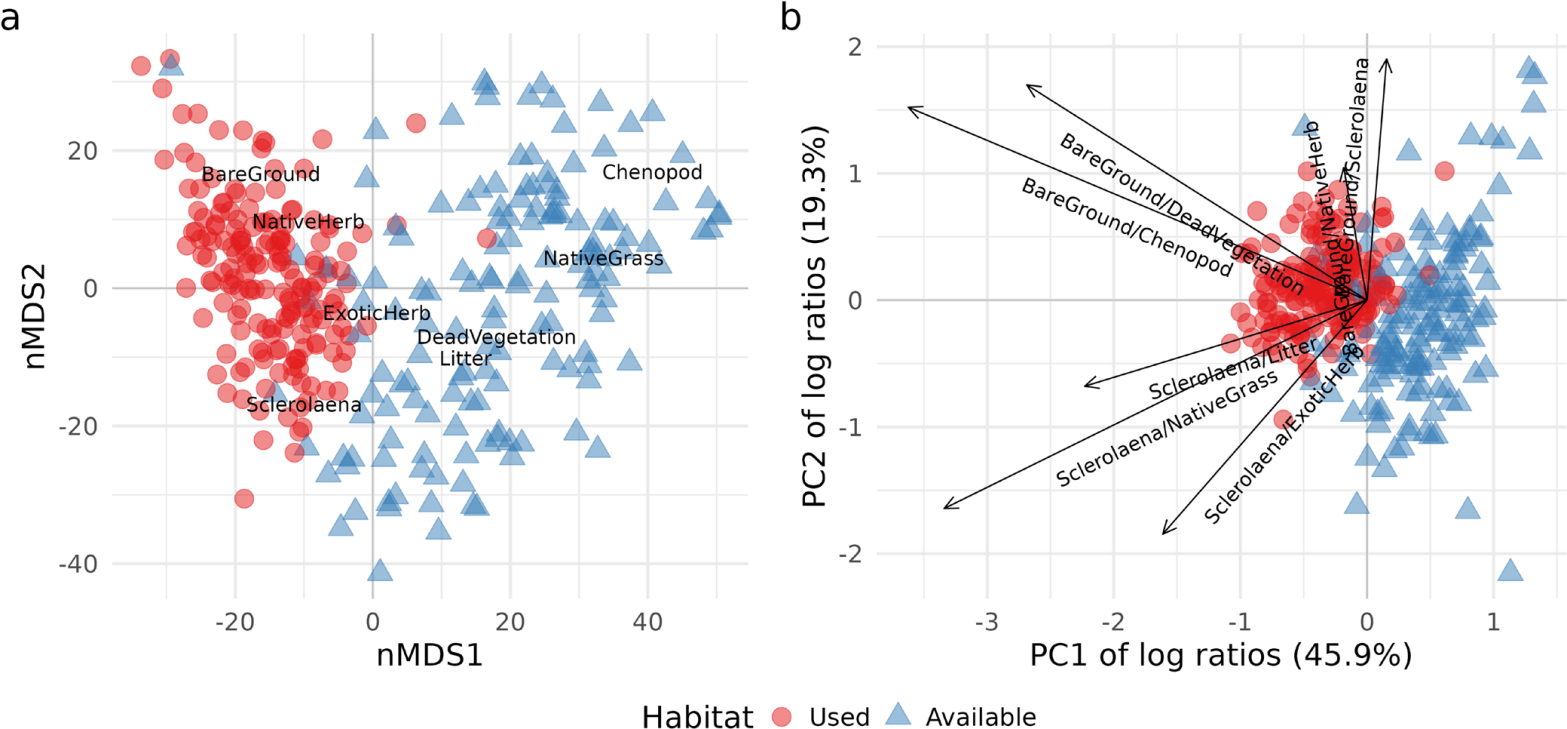
(a) Ordination of used (red circles) and available (blue triangles) habitat via non‐metric multidimensional scaling (nMDS) based on the percent cover of resources in areas of ‘used’ versus ‘available’ habitat. (b) Principal component analysis of log ratios of vegetation cover composition categories measured at sites used (red circles) and unused but available (blue triangles) to Plains‐wanderers.

The nMDS plot revealed a clear separation between used and available habitat (Figure 5). Bare ground, *Sclerolaena* and native forb cover had higher cover in habitat used by Plains‐wanderers. In contrast, higher coverage of Chenopods, native grass, dead vegetation and litter cover was associated with habitat that was available but not used by Plains‐wanderers. Separation of the characteristics of used and available habitat was also clearly evident in the first two dimensions of a PCA of the log ratios of the compositional vegetation cover categories (Figure 5b). The first two principal components accounted for 65% of the total variation in log ratios (45.9% and 19.3% of the variance, respectively). The cover of bare ground relative to Chenopods was the most influential predictor of habitat use, with Plains wanderers using larger amounts of bare ground and selecting against areas with high Chenopod cover (Figure 5b; Table 1). Additional variation in the probability of use was explained by the ratio of bare ground to dead vegetation, again reflecting the avoidance of habitat with dead Chenopods and the use of habitat with higher cover of *Sclerolaena* relative to invasive forbs (Figure 5b; Table 1). Importantly, all log ratio variables ranked higher as independent predictors of habitat use relative to the null model (Appendix 5).

**TABLE 1:** Stepwise selection of log ratios showing the Akaike's information criterion (AIC) and the improvement in AIC (ΔAIC) for each model relative to the model in the row immediately above in the table.

Modelled explanatory variables for log ratios	AIC	ΔAIC
Null	453.2	—
Bare ground versus *Chenopod*	137.3	−315.9
Bare ground versus dead vegetation	131.8	−5.5
*Sclerolaena* versus invasive forb	131.4	−0.4

**FIGURE 6:**
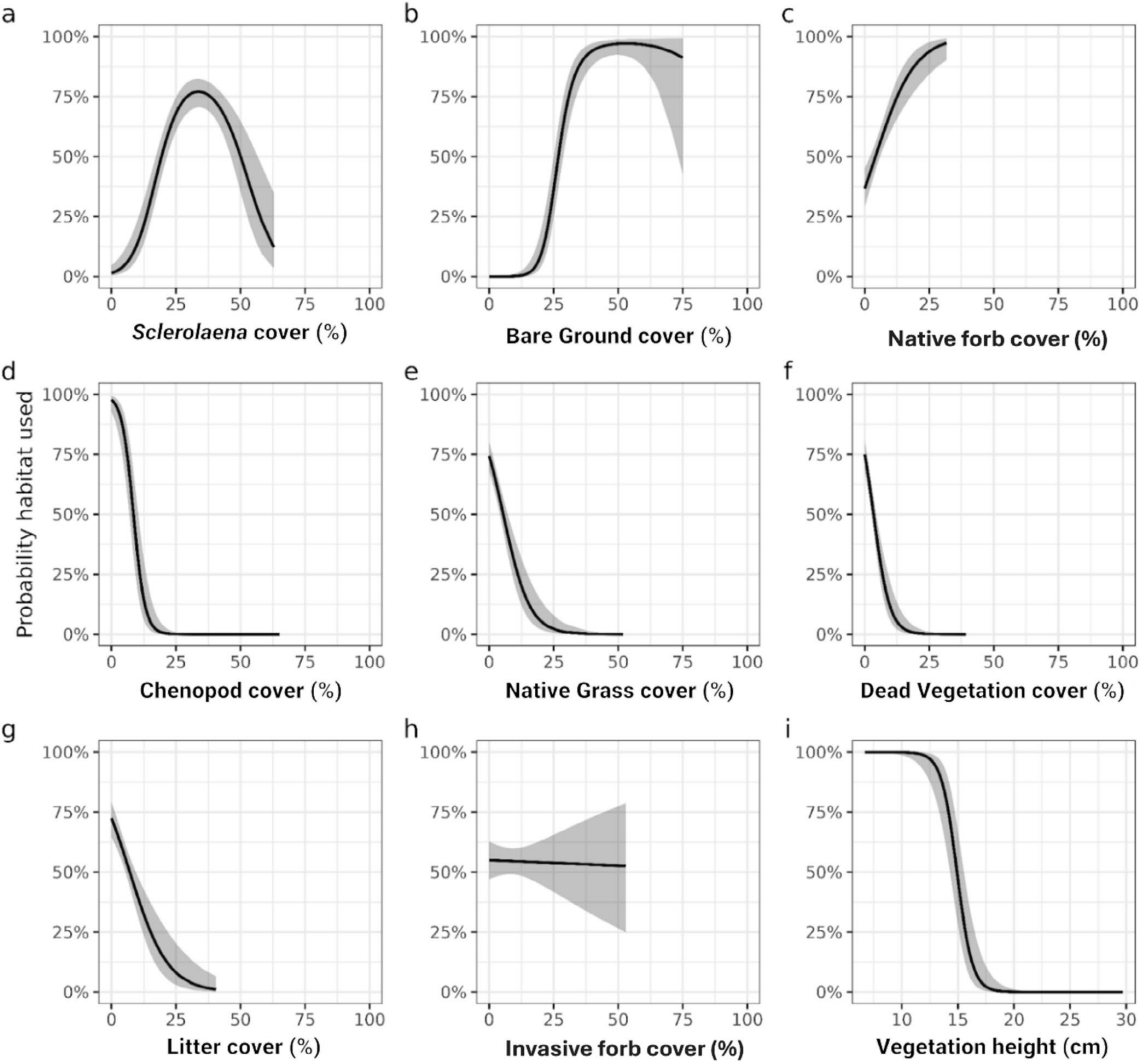
Relationships between probability of habitat use by Plains‐wanderers and the percent cover of habitat features: (a) *Sclerolaena*, (b) bare ground, (c) native forbs, (d) Chenopod, (e) native grasses, (f) dead vegetation, (g) litter, (h) invasive forbs and (i) average vegetation height. The term ‘Chenopod’ was used to refer to all species in the family *Chenopodiaceae*, except the genus ‘*Sclerolaena*,’ which was analysed separately. Solid lines are estimated probabilities from generalised linear mixed‐effects models, and the grey ribbons are 95% confidence intervals.

On average, used habitat consisted of 55% vegetation (SD = 6%), with a statistically supported difference between used and available sites (Figure 6a–g; Appendix 4). In particular, Plains‐wanderers were recorded using habitat that consisted of 32% (SD = 10%) *Sclerolaena* cover, 15% forb cover (10% invasive forb species cover (SD = 8%) and 5% native forb species cover (SD = 4%)), 3% (SD = 3%) Chenopod cover, 3% (SD = 3%) grass cover and 2% (SD = 2%) dead vegetation (Figure 6a–g; Appendices 4 and 5). Used habitat was characterised by 40% bare ground (SD = 11%) and 5% litter (SD = 4%) (Figure 6e,i; Appendix 4). A steep decline in probability of use was recorded in all characteristics when cover exceeded these thresholds (Figure 6). All vegetation categories (Chenopod, bare ground, native grass, dead vegetation, *Sclerolaena*, litter and native forb), with the exception of invasive forb cover, ranked higher than the null model in predicting habitat use (Appendix 6). The proportion of Chenopods present within the site ranked highest as a predictor of Plains‐wanderer habitat use, with the species showing a strong aversion to the habitat type.

**FIGURE 7:**
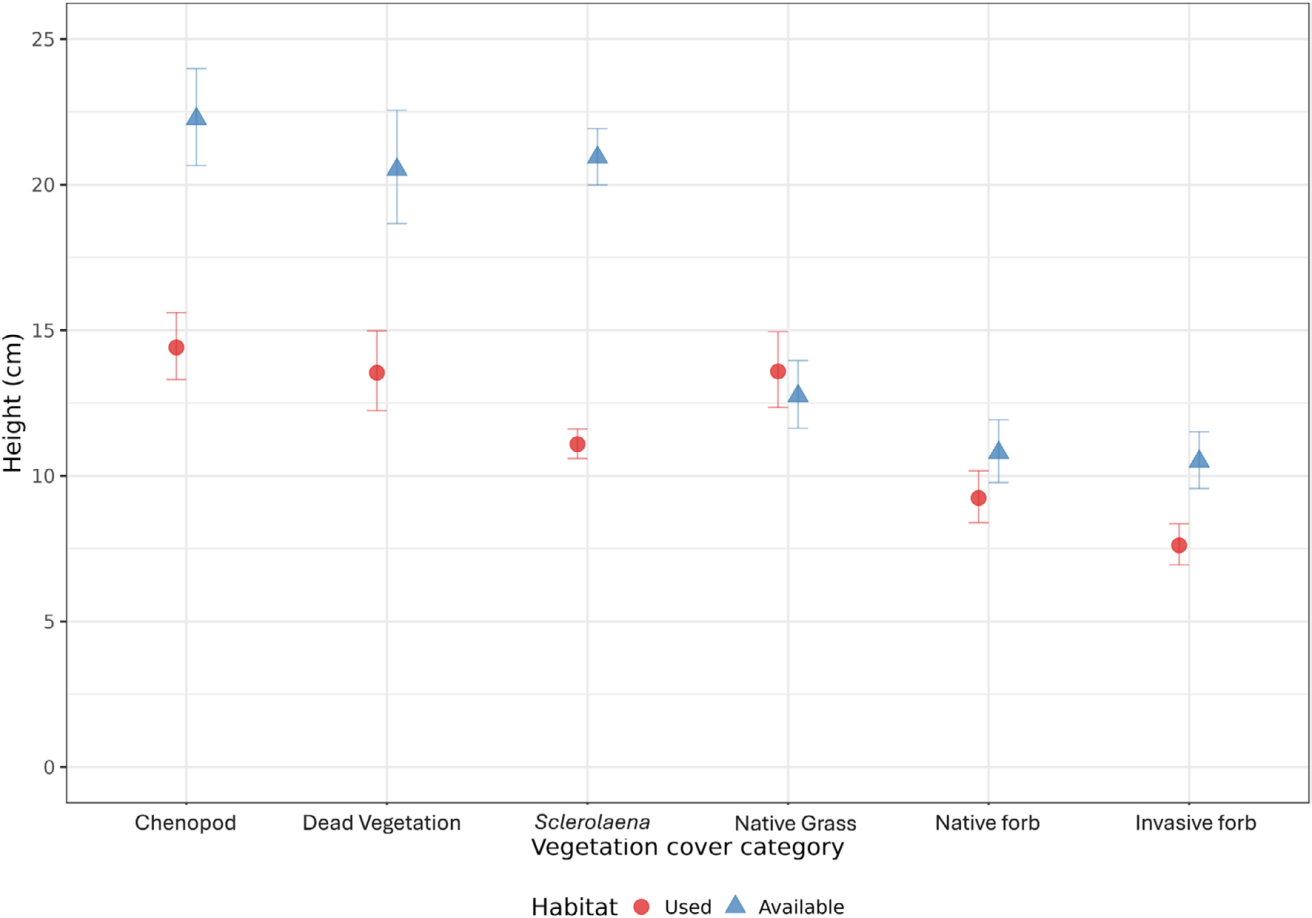
Average height data across vegetation classes with habitat used by Plains‐wanderers during this study shown as red circles, and habitat that was available but not used shown as blue triangles.

Vegetation height was also a key factor in determining the probability of Plains‐wanderer use (Figure 6i), with used habitat measuring 11 cm on average (Appendix 4). The average height of all vegetation categories, except for native grasses, differed between used and available habitats (Appendix 7). Mean height for *Sclerolaena*, Chenopods, native forbs, invasive forbs and dead vegetation in used habitat was shorter in comparison to habitat that was available but not used (Figure 7; Appendix 7).

## 4 Discussion

The long‐held notion that Plains‐wanderers are exclusively ‘grassland specialists’ can now be broadened to incorporate the suitability of low forb‐like Chenopod shrublands, at least at the periphery of the species' extant range. Vegetation communities used by Plains‐wanderers in this study were low Chenopod shrublands, in particular *Sclerolaena divaricata* assemblages. Open vegetation that covered 55% of the habitat, composed mostly of *Sclerolaena* species, with an average height of 11 cm, was a characteristic indicator of Plains‐wanderer habitat use. Our results contrast previous findings in the species stronghold (Baker‐Gabb et al. 1990; Schultz et al. 2017) and identify a vegetation community that has not been previously described as suitable habitat for the species. Habitat use for Plains‐wanderers was more varied than anticipated, with individuals selecting for specific vegetation structures rather than being limited to one vegetation type. This finding was emphasised by the presence of native grasses in the habitats available to Plains‐wanderers, but not in the habitats used by them. The limited use of available native grass habitat suggests that vegetation structure (i.e., habitat openness) and height, rather than the mere presence of specific plant types, are more influential in determining habitat suitability.

While vegetation species recorded in this study varied from other Plains‐wanderer studies, the composition and structure of the habitat are consistent with these previous studies. *Sclerolaena divaricata* is a low‐growing (maximum 50 cm) compactly arranged shrub, characterised by long, thin spines (Brown 2014). It occurs in a community with other species of *Sclerolaena* ( *S. brachyptera* and *S. cuneata* ) and annual forb species. The community is typical of dryland vegetation in semi‐arid South Australia (Jessop and Tolken 1986) and is often associated with disturbance from historic and ongoing grazing practices. As the species is generally unpalatable to livestock, the seed is able to proliferate in good conditions and often comes to dominate pastoral areas (Brown 2014). It is possible that periphery Plains‐wanderer populations are using *Sclerolaena divaricata*, as this association maintains an open habitat structure, similar to the grassland structuring found within the Plains‐wanderers' eastern distribution (Baker‐Gabb 2016; DPIE 2020; Nugent et al. 2023; Parker et al. 2024). The average height of *Sclerolaena* species recorded in this study (11 cm) also aligns with the 15 cm height of grasslands within the core range (Baker‐Gabb 2016). The broad habitat structuring recorded in this study also continues to reflect the structural requirements of Plains‐wanderer habitat identified within the core range, with a similar 50% bare ground, 10% litter and 40% vegetation composition (Baker‐Gabb 2016; DPIE 2020). As such, we suggest that height and composition are more important for predicting Plains‐wanderer occurrence than vegetation community composition. This aligns with broader findings that demonstrate grassland birds responding to structural attributes within their environment (Cingolani et al. 2003; Báez et al. 2013; Antos and Schultz 2020; Nugent et al. 2022). Grassland birds are particularly responsive to the structure of vegetation assemblages to balance foraging, predator avoidance and inter‐ and intraspecific interactions in a relatively open environment (Brennan and Kuvlesky 2005; Winter et al. 2005). As many grassland species display narrow tolerances for structural variation (Howland et al. 2014; Mueller et al. 2008), species composition alone may not be a reliable predictor of habitat use in dynamic or marginal systems.

Regional variation in habitat selection has been recorded for other species, particularly those with broad geographic ranges or occupying heterogeneous landscapes. Habitat composition can vary considerably across space, with habitat use often shaped by a combination of both local and generalised factors (Manning et al. 2007; McAlpine et al. 2008). For example, contrasting patterns in sugar glider ( *Petaurus breviceps* ) occupancy have been observed between Tasmania and mainland Australia (Allen et al. 2018; Goldingay et al. 2020). Similarly, regional preferences have been recorded between populations in the Lesser Spotted Eagle (*Clanga pomarina*) (Väli et al. 2004). These examples highlight a similar pattern to observations in this study, where compositionally different but structurally similar habitats to the previously documented requirements still ensure ecological requirements are supported.

While *Sclerolaena divaricata* communities superficially reflect core range habitat structure, it is also likely that the vegetation association provides unique resources that are beneficial to Plains‐wanderers. This vegetation community supports a variety of invertebrate species, which make up a significant portion of the Plains‐wanderer's diet (Baker‐Gabb 1988). Furthermore, it is likely that the complex, thorny structure of *Sclerolaena divaricata* supports protection from predators.

Despite historic Plains‐wanderer sightings in the South Australian periphery being almost solely recorded in remnant grasslands or within cereal crops surrounding Adelaide (Carpenter 1990), recent sightings have been primarily within the northeast pastoral district, a region that is more typically characterised by the vegetation communities described in this study (Bennett 1983). Owing to the limited historical survey efforts in this region, it remains uncertain whether the species has long persisted in the area or whether its presence reflects a recent shift driven by population fragmentation and altered vegetation conditions. However, the population abundance recorded in this study suggests a potentially stable and established population, which may indicate ecological plasticity in habitat use under changing environmental conditions.

While previous studies have highlighted the specialised nature of Plains‐wanderer habitat use, our results are perhaps not surprising when considering that species range edges are typically characterised by different environmental conditions than core habitats (Gaston 2003). This can be attributed to factors such as reduced resource availability, alternate climatic conditions and different risks associated with predation and disease (Soulé 1973; Hoffmann and Parsons 1997; Holt 2003). These periphery ranges can produce unique selective pressures, which can drive local acclimatisation, such as alternate habitat use (Lesica and Allendorf 1995; Hargreaves and Eckert 2019). Over time, populations may adapt to survive and reproduce in periphery habitats such that they are no longer considered marginal (Kawecki 2008).

Prior predictions suggested that the western periphery of the Plains‐wanderer's range retained only 10% of the known population (Commonwealth 2016), which equates to approximately 25–500 birds based on current population estimates (IUCN 2024). However, this study detected 272 individual Plains‐wanderers in one conservation reserve alone, suggesting the population in this region may be more robust than was previously estimated. This unexpected finding raises the possibility that birds in this study area may be locally habituated to their environment, allowing for greater survival and reproduction despite the periphery location. For instance, tracked Plains‐wanderers maintained fixed home ranges between 1.3 and 113.9 ha (average = 53.6 ha (SD = 52.0 ha)) over the study period. These home‐range sizes are consistent with those recorded in other parts of the species' range (Baker‐Gabb et al. 1990; Commonwealth 2016; Nugent et al. 2022), contrary to prior projections that home ranges would likely be larger at the periphery due to insufficient resources (Commonwealth 2016). The observed home‐range sizes suggest that the species is able to access adequate resources in the peripheral environment, as their spatial behaviour does not reflect the resource scarcity expected in range edge populations (Gill and Wolf 1975; Powell 2000). There is no evidence to suggest that Plains‐wanderers on the periphery of the western distribution have diverged from the core population; however, the persistence of the species and successful breeding at the range edge may suggest a degree of acclimatisation to the western environments. Further investigation is needed to confirm whether these populations are diverging genetically or simply exhibiting adaptive responses to specific ecological conditions. However, the novel evidence of persistence and reproduction provides valuable insights into the potential for edge populations to habituate to new ecological niches, highlighting the evolutionary significance of range edges in shaping species' distributions (Thakur et al. 2018).

## 5 Conclusions

This study provides new insights into the habitat use of an endangered bird at the edge of its distribution, challenging previous assumptions on habitat use. Traditionally considered a grassland specialist, our findings reveal that Plains‐wanderers are using low forb‐like Chenopod shrublands at the range edge. This result is particularly important as it demonstrates the species' ability to thrive in a peripheral habitat, suggesting potential for adaptation and resilience in the face of environmental changes. The persistence of this population at the range edge offers valuable insights into the ecology of peripheral populations, highlighting the importance of peripheral habitats in species conservation. Our findings contribute to the growing body of literature that describes the significance of peripheral populations, which may serve as critical reservoirs of genetic diversity and adaptive potential (Lesica and Allendorf 1995; Channell and Lomolino 2000; Channell 2004; Eckstein et al. 2006; Ledoux et al. 2015). This study also reinforces a cautionary tale: focusing exclusively on a species' habitat use within the core of its range can be a highly limiting factor in species management (Channell and Lomolino 2000). As core habitats often represent the conditions most ideal for a species (Eckert et al. 2008), failing to understand how this changes across a species range can lead to an incomplete understanding of habitat flexibility and the full use of ecological niches across a distribution (Channell and Lomolino 2000). Such limitations may result in inadequate conservation strategies that fail to address habitat needs across the entire range or limit detection possibilities. Therefore, a comprehensive assessment of a species' habitat should include both core and peripheral regions to ensure effective conservation and management practices that account for the full spectrum of habitat use.

Further study on Plains‐wanderer ecology at the range edge is required to investigate if the novel findings are from habitat adaptation and particularly whether these adaptations result from genetic divergence from core populations. Additionally, given that similar habitats are still present throughout the species' historic range, it is possible that additional undetected populations exist, warranting further survey effort. The differences in habitat selection observed in the periphery suggest that broader, more flexible habitat use may be critical for species persistence in marginal areas, a finding particularly important to other range edge populations.

## Author Contributions

**Saskia P. Gerhardy:** conceptualization (lead), data curation (lead), formal analysis (lead), funding acquisition (equal), investigation (lead), methodology (lead), project administration (lead), visualization (lead), writing – original draft (lead), writing – review and editing (lead). **Steven Delean:** conceptualization (supporting), formal analysis (supporting), funding acquisition (supporting), investigation (supporting), methodology (supporting), project administration (supporting), supervision (equal), visualization (supporting), writing – review and editing (equal). **Liberty G. M. Olds:** conceptualization (supporting), investigation (supporting), writing – review and editing (equal). **Graeme Finlayson:** conceptualization (supporting), data curation (supporting), formal analysis (supporting), funding acquisition (equal), investigation (supporting), methodology (supporting), project administration (equal), supervision (equal), writing – review and editing (equal).

## Conflicts of Interest

The authors declare no conflicts of interest.

## Supporting information


**Data S1:** ece371894‐sup‐0001‐supinfo.csv.

**Data S2:** ece371894‐sup‐0002‐supinfo.csv.

**Data S3:** ece371894‐sup‐0003‐supinfo.R.

**Data S4:** ece371894‐sup‐0004‐supinfo.R.

## Data Availability

All the required data are uploaded as Supporting Information.

## Appendix 1 GPS Tracking Parameters of Plains‐Wanderers From September 2022, April 2023 and September 2023

**Table:**

Season/year	Bird ID	Method	Paired (Y/N)	Fixes	Days tracked	Notes
Sept '22	F02	GPS	N	72	20	
Sept '22	F08	GPS	N	5	15	
Sept '22	F10	GPS	N	75	20	
Sept '22	F81	GPS	N	49	17	
Sept '22	F16	GPS	N	74	18	
Sept '22	F18	GPS	N	72	18	
Sept '22	F79	GPS	N	42	15	
Sept '22	F77	GPS	N	23	18	
Sept '22	F78	GPS	N	52	17	
Sept '22	F75	GPS	N	59	16	
April '23	F20	GPS	N	58	15	
April '23	F11	GPS	N	25	6	Deceased
April '23	F13	GPS	N	19	5	Deceased
April '23	M08	VHF	N	18	29	
April '23	M02	VHF	N	19	31	
April '23	M07	VHF	N	13	20	
April '23	M01	VHF	N	19	31	
April '23	M03	VHF	N	18	31	
Sept '23	F54	GPS	N	18	7	Deceased
Sept '23	F56	GPS	N	29	23	
Sept '23	F58	GPS	N	46	24	
Sept '23	M53	VHF	N	13	19	
Sept '23	M57	VHF	N	13	19	
Sept '23	M59	VHF	N	15	18	
Sept '23	F60	VHF	N	11	18	
Sept '23	M65	VHF	N	12	15	
Sept '23	F66	VHF	M67	17	15	
Sept '23	M67	VHF	F66	17	15	
Average				37 (SD = 25)	19 (SD = 7)	

*Note:* Bird ID Refers To the Sex of the Bird (M = Male and F = Female) and ABBBS Band Number.

## Appendix 2 Home‐Range Size (Hectares) for Plains‐Wanderers Across the Tracking Period as Calculated by Minimum Convex Polygon (MCP), 95% Kernel Density Estimates and 50% Kernel Density Estimate

**Table:**

Season/year	Bird ID	MCP (Ha)	Kernel density estimate		Autocorrelated kernel density estimates	
			95% kernel density (ha)	50% Kernel density (ha)	95% kernel density (ha)	50% Kernel density (ha)
Sept '22	F02	115.7	113.9	29.8	116.5	32.1
Sept '22	F08	26.9	43.2	10.3	9.1	2.2
Sept '22	F10	81.9	185	51.2	380.4	95.3
April '23	F11	11.1	67.4	16.5	67.8	16.7
April '23	F13	7.1	21.3	3.4	8.2	2.4
Sept '22	F16	21.1	18.2	3.6	11.7	2.4
Sept '22	F18	10.9	18.6	5	14.9	4.1
April '23	F20	19.2	19.5	5	16.9	4.5
Sept '23	F54	2.9	15.8	4.4	na	na
Sept '23	F56	3.2	23.1	6.5	17.8	4.7
Sept '23	F58	16	60.1	16.1	na	na
Sept '23	F60	11.6	42.6	9.8	47.5	12.5
Sept '23	F66&M67	24.8	3	0.8	na	na
Sept '22	F75	56.5	59.7	15.2	18.1	4.6
Sept '22	F77	3.9	35.5	6.9	nil	nil
Sept '22	F78	15.1	9.8	2.3	8	1.8
Sept '22	F79	8.4	15.3	3.1	na	na
Sept '22	F81	60.9	52.6	8	27.8	4.3
April '23	M01	12.7	36.1	10	na	na
April '23	M02	6.5	20.8	6.3	na	na
April '23	M03	34.3	125.8	29	na	na
April '23	M07	34.3	166.7	36.7	na	na
April '23	M08	35.9	164.2	42	na	na
Sept '23	M53	8.9	19.3	4.5	na	na
Sept '23	M57	34.5	71.7	12.2	na	na
Sept '23	M59	5.73	14.4	3.2	na	na
Sept '23	M65	8.3	25.3	6.2	na	na
Average		25.1 (SD = 19.8)	53.6 (SD = 51.5)	12.9 (SD = 13.1)	58.6 (SD = 74.5)	14.4 (SD = 18.7)

*Note:* As a comparison, Continuous Time Movement Models (CTMM) were also applied to estimate autocorrelated kernel density estimates (AKDEs). Variograms were fit for each individual and used to guide model selection. The best‐fitting models were then used to compute AKDEs using the ctmm package in R (Fleming and Calabrese 2022). In cases where sufficient location data were available, AKDE values aligned closely with 95% KDE estimates, supporting the robustness of the KDE‐based approach. AKDEs could not be computed for males due to limited datasets. Given data collection was not optimised for full home‐range delineation, MCPs were retained as the primary metric for defining used habitat. However, the CTMM results did highlight the potential for broader space use beyond observed locations, which helped guide the placement of paired ‘available’ habitat surveys outside likely use areas.

## Appendix 4 Results From Kruskal–Wallis Rank Sum Test (*p*) Including Mean Percent Cover and Standard Deviation (±SD) for the Percent Cover of Bare Ground, Native Grass, *Sclerolaena* spp., Chenopodiaceae, Native Forbs, Invasive Forbs, Dead Vegetation, Litter and Height Between Used and Available Habitat

**Table:**

	Mean % cover for used (±SD)	Mean % cover for available (±SD)	DF	P value
Bare ground	40.5 (±11.0)	17.4 (±10.6)	1	2.2e−16
Native Grass	2.58 (±2.87)	9.94 (±11.9)	1	1.028e−08
*Sclerolaena* spp.	31.6 (±9.62)	22.7 (±15.7)	1	1.56e−08
*Chenopodiaceae* spp.	2.86 (±3.36)	22.8 (±14.7)	1	2.2e−16
Native Forb	4.60 (±4.44)	8.41 (±7.62)	1	0.0002863
Invasive forb	9.97 (±8.03)	8.98 (±8.53)	1	0.1819
Dead vegetation	1.70 (±2.20)	5.59 (±6.30)	1	1.084e−11
Litter	4.60 (±4.44)	8.41 (±7.62)	1	3.914e−07
Average height	10.7 (±2.00)	20.1 (±3.76)	1	2.2e−16

*Note: p*‐values less than 0.05 were considered significant.

## Appendix 5 Results of Akaike Information Criterion Ranking Generalised Linear Mixed Model Selection of Habitat Features That Best Explain Plains‐Wanderer Habitat Use

**Table:**

Modelled explanatory variables	Df	ΔAIC	AIC_c_ Weight
Bare Ground versus Chenopod	3	0.00	1
Bare Ground versus Dead Vegetation	3	195.4	0
*Sclerolaena* versus Native Grass	3	239.7	0
*Sclerolaena* versus Litter	3	247.1	0
*Sclerolaena* versus Invasive Forb	3	298.1	0
Bare Ground versus *Sclerolaena*	3	311.0	0
Bare Ground versus Native Forb	3	315.5	0
Null	2	315.8	0

*Note:* Each model is a log ratio explanatory variable with individual bird identification specified as a random effect. ΔAIC is the difference in AIC_c_ from the minimum AIC_c_ model. The term ‘Chenopod’ was used to refer to all species in the family *Chenopodiaceae*, except the genus ‘*Sclerolaena*’ which was analysed separately.

## Appendix 6 Results of Akaike Information Criterion Ranking Model Selection of Habitat Features That Best Explain Plains‐Wanderer Habitat Use

**Table:**

Modelled explanatory variables	ΔAIC	AIC_c_ weight
Percentage of *Chenopod* cover	0.00	1
Percentage of bare ground cover	21.10	0
Percentage of native grass cover	184.7	0
Percentage of dead vegetation cover	196.9	0
Percentage of *Sclerolaena* cover	229.5	0
Percentage of litter cover	232.1	0
Percentage of native forb cover	237.1	0
Null	266.6	0
Percentage of invasive forb cover	268.7	0

*Note:* Each model is a generalised linear mixed model with individual bird identification as a random effect. The null model does not include any vegetation openness metric. ΔAIC is the difference in AIC_c_ from the minimum AIC_c_ model. The term ‘Chenopod’ was used to refer to all species in the family *Chenopodiaceae*, except the genus ‘*Sclerolaena’* which was analysed separately.

## Appendix 7 Results of Akaike Information Criterion Ranking Model Selection of Vegetation Height

**Table:**

Response variable	Explanatory variable	Df	ΔAIC	AIC_c_ weight
*Sclerolaena*	Used versus available	4	0.0	1
	Constant	3	246.8	0
*Chenopod*	Used versus available	4	0.0	1
	Constant	3	65.18	0
Native grass	Constant	3	0.0	0.6
	Used versus available	4	0.6	0.4
Native forb	Used versus available	4	0.0	0.9
	Constant	3	5.3	0.1
Invasive forb	Used versus available	4	0.0	1
	Constant	3	46.3	0
Dead vegetation	Used versus available	4	0.0	1
	Constant	3	47.1	0

*Note:* The difference in height of vegetation characteristics (*Sclerolaena*, *Chenopod*, Native grasses, native forbs, invasive forbs and dead vegetation) between used and available habitat was ranked against the null model. In all instances besides native grasses, the difference between used and available habitat was preferred over the null model. Order of the explanatory variable is used to identify which model is supported. ΔAIC is the difference in AIC_c_ from the minimum AIC_c_ model. The term ‘Chenopod’ was used to refer to all species in the family *Chenopodiaceae*, except the genus ‘*Sclerolaena’* which was analysed separately.

## References

[ece371894-bib-0001] Aide, T. M. , C. Corrada‐Bravo , M. Campos‐Cerqueira , C. Milan , G. Vega , and R. Alvarez . 2013. “Real‐Time Bioacoustics Monitoring and Automated Species Identification.” PeerJ 1: e103. 23882441 10.7717/peerj.103 PMC3719130

[ece371894-bib-0002] Allen, M. , M. H. Webb , F. Alves , R. Heinsohn , and D. Stojanovic . 2018. “Occupancy Patterns of the Introduced, Predatory Sugar Glider in Tasmanian Forests.” Austral Ecology 43, no. 4: 470–475.

[ece371894-bib-0003] Antos, M. , and N. L. Schultz . 2020. “Climate‐Mediated Changes to Grassland Structure Determine Habitat Suitability for the Critically Endangered Plains‐Wanderer (*Pedionomus torquatus*).” Emu 120: 2–10.

[ece371894-bib-0004] Báez, S. , S. L. Collins , W. T. Pockman , J. E. Johnson , and E. E. Small . 2013. “Effects of Experimental Rainfall Manipulations on Chihuahuan Desert Grassland and Shrubland Plant Communities.” Oecologia 172: 1117–1127. 23263528 10.1007/s00442-012-2552-0

[ece371894-bib-0005] Baker‐Gabb, D. 2016. Managing Grasslands for the Plains‐Wanderer Handbook. Elanus consulting.

[ece371894-bib-0006] Baker‐Gabb, D. , M. Antos , and G. Brown . 2016. “Recent Decline of the Critically Endangered Plains‐Wanderer ( *Pedionomus torquatus* ), and the Application of a Simple Method for Assessing Its Cause: Major Changes in Grassland Structure.” Ecological Management & Restoration 17, no. 3: 235–242.

[ece371894-bib-0007] Baker‐Gabb, D. J. 1988. “The Diet and Foraging Behaviour of the Plains‐Wanderer *Pedionomus torquatus* .” Emu 88: 115–118.

[ece371894-bib-0008] Baker‐Gabb, D. J. 1990. “An Annotated List of Records of Plains‐Wanderers Pedionomus Torquatus, 1980–89.” Australian Bird Watcher 13: 249–252.

[ece371894-bib-0009] Baker‐Gabb, D. J. 1998. “Native Grasslands and the Plains‐Wanderer.” Birds Australia Conservation Statement No. 1. Wingspan 8: 1–8.

[ece371894-bib-0011] Baker‐Gabb, D. J. , J. S. Benshemesh , and P. N. Maher . 1990. “A Revision of the Distribution, Status and Management of the Plains‐Wanderer *Pedionomus torquatus* .” Emu—Austral Ornithology 90: 161–168.

[ece371894-bib-0013] Bartoń, K. 2022. “_MuMIn: Multi‐Model Inference_.” R Package Version 1.47.1. https://CRAN.R‐project.org/package=MuMIn.

[ece371894-bib-0014] Bellchambers, K. , and D. J. Baker‐Gabb . 2006. A Survey of Plains‐Wanderers and Thick‐Billed Grasswrens in the North‐East Pastoral Zone of South Australia. Report to South Australian Arid Lands Natural Resources Management Board. https://cdn.environment.sa.gov.au/landscape/docs/saal/plains‐wanderers‐and‐thick‐billed‐grasswrens‐2009‐survey‐gen.pdf.

[ece371894-bib-0015] Bennett, S. 1983. “A Review of the Distribution, Status and Biology of the Plains‐Wanderer *Pedionomus torquatus* Gould.” Emu 83: 1–11.

[ece371894-bib-0016] BirdLife International . 2022. “ *Pedionomus torquatus* . The IUCN Red List of Threatened Species 2022: e.T22693049A212570062.” Accessed December 12, 2024. 10.2305/IUCN.UK.2022-1.RLTS.T22693049A212570062.en.

[ece371894-bib-0018] BOM . 2024. Australian Bureau of Meteorology. Climate Data Online. Accessed March 15, 2024. http://www.bom.gov.au/climate/data/kotta.

[ece371894-bib-0019] Brennan, L. A. , and W. P. Kuvlesky Jr. 2005. “North American Grassland Birds: An Unfolding Conservation Crisis?” Journal of Wildlife Management 69, no. 1: 1–13.

[ece371894-bib-0020] Brooks, M. E. , K. Kristensen , K. J. Van Benthem , et al. 2017. “glmmTMB Balances Speed and Flexibility Among Packages for Zero‐Inflated Generalized Linear Mixed Modeling.” R Journal 9, no. 2: 378–400.

[ece371894-bib-0095] Brown, N. F. 2014. Simply Saltbush: A Guide to the Identification and Uses of Chenopods in the Murray Region of SA. Mid Murray Local Action Planning Committee Incorporated.

[ece371894-bib-0021] Carpenter, G. 1990. “The Status and Distribution of the Plains‐ Wanderer in South Australia.” Unpublished Paper.

[ece371894-bib-0098] Channell, R. 2004. “The Conservation Value of Peripheral Populations: The Supporting Science.” In Proceedings of the Species at Risk 2004 Pathways to Recovery Conference, 1–17. Species at Risk 2004 Pathways to Recovery Conference Organizing Committee.

[ece371894-bib-0022] Channell, R. , and M. V. Lomolino . 2000. “Dynamic Biogeography and Conservation of Endangered Species.” Nature 403, no. 6765: 84–86. 10638757 10.1038/47487

[ece371894-bib-0023] Cingolani, A. M. , M. R. Cabido , D. Renison , and V. Solís Neffa . 2003. “Combined Effects of Environment and Grazing on Vegetation Structure in Argentine Granite Grasslands.” Journal of Vegetation Science 14: 223–232.

[ece371894-bib-0024] Commonwealth of Australia . 2016. National Recovery Plan for the Plains‐Wanderer ( *Pedionomus torquatus* ). Commonwealth of Australia.

[ece371894-bib-0025] Dawlings, F. M. , M. Humphrey , D. T. Nugent , and R. H. Clarke . 2024. “Thermal Scanners Versus Spotlighting: New Opportunities for Monitoring Threatened Small Endotherms.” Austral Ecology 49, no. 5: e13544.

[ece371894-bib-0026] De Pietri, V. L. , R. P. Scofield , A. J. Tennyson , S. J. Hand , and T. H. Worthy . 2016. “Wading a Lost Southern Connection: Miocene Fossils From New Zealand Reveal a New Lineage of Shorebirds (Charadriiformes) Linking Gondwanan Avifaunas.” Journal of Systematic Palaeontology 14: 603–616.

[ece371894-bib-0028] DPIE . 2020. Plains‐Wanderer Habitat Management Guide. State of New South Wales Department of Planning, Industry, and Environment, Sydney, Australia.

[ece371894-bib-0029] Dudaniec, R. Y. , S. F. Spear , J. S. Richardson , and A. Storfer . 2012. “Current and Historical Drivers of Landscape Genetic Structure Differ in Core and Peripheral Salamander Populations.” PLoS One 7, no. 5: e36769. 22590604 10.1371/journal.pone.0036769 PMC3349670

[ece371894-bib-0030] Eckert, C. G. , K. E. Samis , and S. C. Lougheed . 2008. “Genetic Variation Across Species' Geographical Ranges: The Central–Marginal Hypothesis and Beyond.” Molecular Ecology 17, no. 5: 1170–1188. 18302683 10.1111/j.1365-294X.2007.03659.x

[ece371894-bib-0099] Eckstein, R. L. , R. A. O’neill , J. Danihelka , A. Otte , and W. Köhler . 2006. “Genetic Structure Among and Within Peripheral and Central Populations of Three Endangered Floodplain Violets.” Molecular Ecology 15, no. 9: 2367–2379. 16842412 10.1111/j.1365-294X.2006.02944.x

[ece371894-bib-0100] ESRI . 2011. ArcGIS Desktop: Release 10. Environmental Systems Research Institute.

[ece371894-bib-0031] Fleming, C. H. , and J. M. Calabrese . 2022. “ctmm: Continuous‐Time Movement Modeling.” Version 1.1.0. https://CRAN.R‐project.org/package=ctmm.

[ece371894-bib-0032] Foreman, P. W. 2010. “Recovery of the Northern Plains Grassland Community—An Overview.” Proceedings of the Royal Society of Victoria 122, no. 2: 92–99.

[ece371894-bib-0034] Gaston, K. J. 2003. The Structure and Dynamics of Geographic Ranges. Oxford University Press.

[ece371894-bib-0035] Gill, F. B. , and L. L. Wolf . 1975. “Economics of Feeding Territoriality in the Golden‐Winged Sunbird.” Ecology 56: 333–345.

[ece371894-bib-0036] Goldingay, R. L. , D. G. Quin , O. Talamo , and J. Mentiplay‐Smith . 2020. “Nest Box Revealed Habitat Preferences of Arboreal Mammals in Box‐Ironbark Forest.” Ecological Management & Restoration 21, no. 2: 131–142.

[ece371894-bib-0037] Greenacre, M. 2019. “Variable Selection in Compositional Data Analysis Using Pairwise Logratios.” Mathematical Geosciences 51, no. 5: 649–682.

[ece371894-bib-0038] Greenacre, M. 2021. “Compositional Data Analysis.” Annual Review of Statistics and Its Application 8, no. 1: 271–299.

[ece371894-bib-0039] Hampe, A. , and R. J. Petit . 2005. “Conserving Biodiversity Under Climate Change: The Rear Edge Matters.” Ecology Letters 8, no. 5: 461–467. 21352449 10.1111/j.1461-0248.2005.00739.x

[ece371894-bib-0040] Hargreaves, A. L. , and C. G. Eckert . 2019. “Local Adaptation Primes Cold‐Edge Populations for Range Expansion but Not Warming‐Induced Range Shifts.” Ecology Letters 22, no. 1: 78–88. 30411457 10.1111/ele.13169

[ece371894-bib-0041] Harrington, G. N. , P. N. Maher , and D. J. Baker‐Gabb . 1988. “The Biology of the Plains‐Wanderer *Pedionomus torquatus* on the Riverine Plain of New South Wales During and After Drought.” Corella 12: 7–13.

[ece371894-bib-0042] Harris, S. , W. J. Cresswell , P. G. Forde , W. J. Trewhella , T. Woollard , and S. Wray . 1990. “Home‐Range Analysis Using Radio‐Tracking Data—A Review of Problems and Techniques Particularly as Applied to the Study of Mammals.” Mammal Review 20, no. 2–3: 97–123.

[ece371894-bib-0043] Hoffmann, A. A. , and P. A. Parsons . 1997. Extreme Environmental Change and Evolution. Cambridge University Press.

[ece371894-bib-0044] Holt, R. D. 2003. “On the Evolutionary Ecology of Species' Ranges.” Evolutionary Ecology Research 5, no. 2: 159–178.

[ece371894-bib-0045] Howland, B. , D. Stojanovic , I. J. Gordon , A. D. Manning , D. Fletcher , and D. B. Lindenmayer . 2014. “Eaten out of House and Home: Impacts of Grazing on Ground‐Dwelling Reptiles in Australian Grasslands and Grassy Woodlands.” PLoS One 9, no. 12: e105966. 25501680 10.1371/journal.pone.0105966 PMC4263405

[ece371894-bib-0097] IUCN . 2024. “International Union for Conservation of Nature.” https://datazone.birdlife.org/species/factsheet/plains‐wanderer‐pedionomus‐torquatus. 10.1098/rsta.2023.0053 38342209

[ece371894-bib-0046] Jenkins, S. H. 1981. “Common Patterns in Home Range‐Body Size Relationships of Birds and Mammals.” American Naturalist 118, no. 1: 126–128.

[ece371894-bib-0096] Jessop, J. P. , and H. R. Tolken . 1986. The Flora of South Australia. Government of South Australia Printer.

[ece371894-bib-0047] Jetz, W. , G. H. Thomas , J. B. Joy , D. W. Redding , K. Hartmann , and A. O. Mooers . 2014. “Global Distribution and Conservation of Evolutionary Distinctness in Birds.” Current Biology 24, no. 9: 919–930. 24726155 10.1016/j.cub.2014.03.011

[ece371894-bib-0048] Kawecki, T. J. 2008. “Adaptation to Marginal Habitats.” Annual Review of Ecology, Evolution, and Systematics 39, no. 1: 321–342.

[ece371894-bib-0050] Kie, J. G. , J. Matthiopoulos , J. Fieberg , et al. 2010. “The Home‐Range Concept: Are Traditional Estimators Still Relevant With Modern Telemetry Technology?” Philosophical Transactions of the Royal Society B: Biological Sciences 365, no. 1550: 2221–2231. 10.1098/rstb.2010.0093 PMC2894967 20566499

[ece371894-bib-0052] Ledoux, J. B. , D. Aurelle , N. Bensoussan , C. Marschal , J. P. Féral , and J. Garrabou . 2015. “Potential for Adaptive Evolution at Species Range Margins: Contrasting Interactions Between Red Coral Populations and Their Environment in a Changing Ocean.” Ecology and Evolution 5, no. 6: 1178–1192. 25859324 10.1002/ece3.1324 PMC4377262

[ece371894-bib-0053] Lesica, P. , and F. W. Allendorf . 1995. “When Are Peripheral Populations Valuable for Conservation?” Conservation Biology 9, no. 4: 753–760.

[ece371894-bib-0054] Lowe, K. W. 1989. The Australian Bird Bander's Manual. Australian Bird and Bat Banding Schemes, Australian National Parks and Wildlife Service.

[ece371894-bib-0056] Manning, A. D. , D. B. Lindenmayer , S. C. Barry , and H. A. Nix . 2007. “Large‐Scale Spatial and Temporal Dynamics of the Vulnerable and Highly Mobile Superb Parrot.” Journal of Biogeography 34, no. 2: 289–304.

[ece371894-bib-0057] Marchant, S. , and P. J. Higgins . 1993. Handbook of Australian, New Zealand and Antarctic Birds. Volume 2. Raptors to Lapwings. Oxford University Press.

[ece371894-bib-0058] McAlpine, C. A. , J. R. Rhodes , M. E. Bowen , et al. 2008. “Can Multiscale Models of Species' Distribution Be Generalized From Region to Region? A Case Study of the Koala.” Journal of Applied Ecology 45, no. 2: 558–567.

[ece371894-bib-0063] Mueller, T. , K. A. Olson , T. K. Fuller , G. B. Schaller , M. G. Murray , and P. Leimgruber . 2008. “In Search of Forage: Predicting Dynamic Habitats of Mongolian Gazelles Using Satellite‐Based Estimates of Vegetation Productivity.” Journal of Applied Ecology 45: 649–658.

[ece371894-bib-0101] New South Wales National Parks and Wildlife Service . 2002. Plains‐Wanderer (Pedionomus torquatus) Recovery Plan. New South Wales National Parks and Wildlife Service.

[ece371894-bib-0064] Nugent, D. T. , D. J. Baker‐Gabb , P. Green , et al. 2022. “Multi‐Scale Habitat Selection by a Cryptic, Critically Endangered Grassland Bird—The Plains‐Wanderer ( *Pedionomus torquatus* ): Implications for Habitat Management and Conservation.” Austral Ecology 47, no. 3: 698–712.

[ece371894-bib-0065] Nugent, D. T. , D. J. Baker‐Gabb , and J. W. Morgan . 2023. “Can Switches in Disturbance Type Improve Habitat for Grassland Birds in Semi‐Arid Grasslands of South‐Eastern Australia?” Austral Ecology 48, no. 8: 2108–2125.

[ece371894-bib-0066] Oksanen, J. , R. Kindt , P. Legendre , et al. 2007. “The Vegan Package: Community Ecology Package.” R Package Version, 1, 1–190.

[ece371894-bib-0067] Olson, S. L. , and D. W. Steadman . 1981. “The Relationships of the Pedionomidae (Aves, Charadriiformes).” Smithsonian Contributions to Zoology 337: 1–25.

[ece371894-bib-0068] Parker, D. G. 2009. “Surveys of the Vertebrate Fauna in Native Grasslands of the Riverine Plain, New South Wales.” Victorian Naturalist 126, no. 4: 128–134.

[ece371894-bib-0069] Parker, D. G. , M. Cameron , C. E. Gordon , and M. Letnic . 2024. “Habitat Structure and an Introduced Predator Limit the Abundance of an Endangered Ground‐Nesting Bird.” Ecological Applications 34: e3046. 39373309 10.1002/eap.3046 PMC11610650

[ece371894-bib-0073] Playfair, R. M. , and A. C. Robinson , eds. 1997. A Biological Survey of the North Olary Plains, South Australia, 1995–1997. Department of Environment and Natural Resources.

[ece371894-bib-0074] Powell, R. A. 2000. “Animal Home Ranges and Territories and Home Range Estimators.” Research Techniques in Animal Ecology: Controversies and Consequences 442: 65–110.

[ece371894-bib-0075] R Core Team . 2022. R: A Language and Environment for Statistical Computing. R Foundation for Statistical Computing, Vienna, Austria. https://www.R‐project.org/.

[ece371894-bib-0078] Rosenzweig, M. L. 1981. “A Theory of Habitat Selection.” Ecology 62, no. 2: 327–335.

[ece371894-bib-0079] Rowe, K. M. , K. E. Selwood , D. Bryant , and D. Baker‐Gabb . 2023. “Acoustic Surveys Improve Landscape‐Scale Detection of a Critically Endangered Australian Bird, the Plains‐Wanderer (*Pedionomus torquatus*).” Wildlife Research 51. 10.1071/WR22187.

[ece371894-bib-0080] Rowe, K. M. C. , and S. Balasubramaniam . 2020. Plains‐Wanderer Acoustic Monitoring: Song Meter Detection Space. Victorian Department of Environment, Land, Water and Planning Report.

[ece371894-bib-0081] Samuel, M. D. , D. J. Pierce , and E. O. Garton . 1985. “Identifying Areas of Concentrated Use Within the Home Range.” Journal of Animal Ecology 54: 711–719.

[ece371894-bib-0082] Schultz, N. , M. Keatley , M. Antos , et al. 2017. “The Golf Ball Method for Rapid Assessment of Grassland Structure.” Ecological Management & Restoration 18: 134–140.

[ece371894-bib-0083] Sibley, C. G. , J. E. Ahlquist , and B. L. Monroe Jr. 1988. “A Classification of the Living Birds of the World Based on DNA‐DNA Hybridization Studies.” Auk 105, no. 3: 409–423.

[ece371894-bib-0085] Soulé, M. 1973. “The Epistasis Cycle: A Theory of Marginal Populations.” Annual Review of Ecology and Systematics 4: 165–187.

[ece371894-bib-0086] Thakur, M. , E. W. Schättin , and W. J. McShea . 2018. “Globally Common, Locally Rare: Revisiting Disregarded Genetic Diversity for Conservation Planning of Widespread Species.” Biodiversity and Conservation 27, no. 11: 3031–3035.

[ece371894-bib-0087] Väli, Ü. , R. Treinys , and A. Lõhmus . 2004. “Geographical Variation in Macrohabitat Use and Preferences of the Lesser Spotted Eagle *Aquila pomarina* .” Ibis 146, no. 4: 661–671.

[ece371894-bib-0089] Wainwright, P. C. 1994. “Functional Morphology as a Tool in Ecological Research.” In Ecological Morphology: Integrative Organismal Biology, edited by P. C. Wainwright and S. M. Reilly , 42–59. University of Chicago Press.

[ece371894-bib-0090] White, A. , B. Sparrow , E. Leitch , et al. 2012. AusPlots Rangelands. University of Adelaide Press.

[ece371894-bib-0091] Wiens, J. A. 1984. “Resource Systems, Populations, and Communities.” In A New Ecology: Novel Approaches to Interactive Systems, edited by P. W. Price , C. N. Slobodchikoff , and W. S. Gaud , 397–436. Wiley‐Interscience.

[ece371894-bib-0092] Willems, E. P. , and R. A. Hill . 2009. “Predator‐Specific Landscapes of Fear and Resource Distribution: Effects on Spatial Range Use.” Ecology 90, no. 2: 546–555. 19323238 10.1890/08-0765.1

[ece371894-bib-0093] Winter, M. , D. H. Johnson , and J. A. Shaffer . 2005. “Variability in Vegetation Effects on Density and Nesting Success of Grassland Birds.” Journal of Wildlife Management 69, no. 1: 185–197.

[ece371894-bib-0094] Worton, B. J. 1989. “Kernel Methods for Estimating the Utilization Distribution in Home‐Range Studies.” Ecology 70, no. 1: 164–168.

